# Liver immunology: new insights from cutting-edge technologies

**DOI:** 10.1038/s41423-025-01342-2

**Published:** 2025-09-22

**Authors:** Adrien Guillot, Bin Gao

**Affiliations:** 1 Charité—Universitätsmedizin Berlin, Department of Hepatology & Gastroenterology, Campus Virchow-Klinikum and Campus Charité Mitte, Berlin, Germany; 2 Laboratory of Liver Diseases, National Institute on Alcohol Abuse and Alcoholism, National Institutes of Health, Rockville, MD USA

**Keywords:** Immunology, Cell signalling


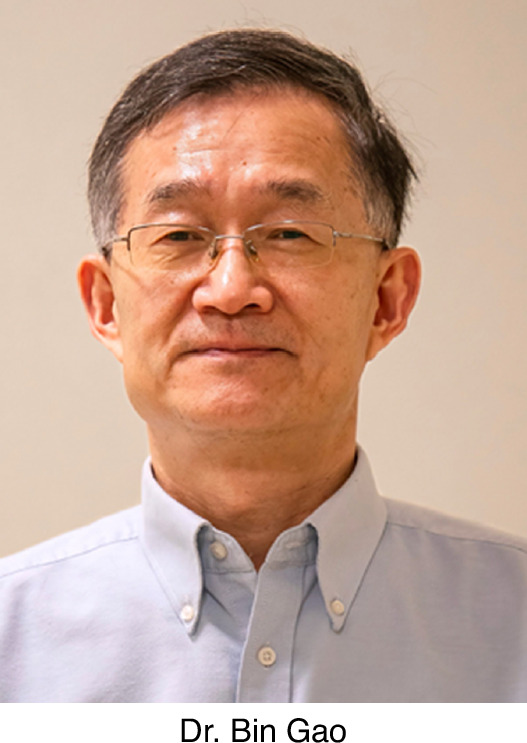

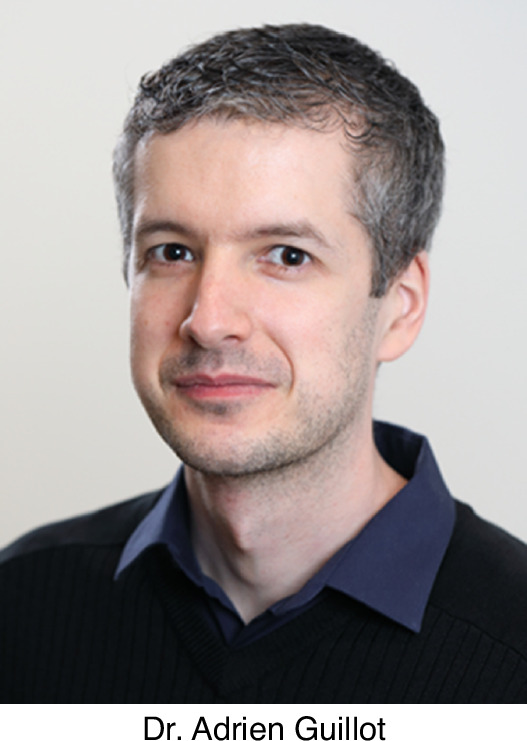
In this article collection, well-established and rising leaders in research related to liver immunology provide their views on the most recent findings in the field. On the basis of comprehensive literature reviews and in-depth analyses of emerging concepts in hepatology, we anticipate that the third edition of the Special Issue “Liver Immunology” will serve as a significant resource. It aims not only to consolidate the latest advancements in the pathophysiology and treatment of liver diseases but also to present expert perspectives on current and evolving trends in the field. This special issue also includes one original research article, which illustrates the application of recent cutting-edge technologies to study macrophages in diseased livers [1]. As emphasized in this collection, many groundbreaking discoveries over the past five years have been facilitated by the increasing adoption of advanced technologies, notably single-cell and single-nucleus transcriptomics, intravital imaging, multiplex immunofluorescence, and the accompanying analytical software tools.

**Fig. 1:**
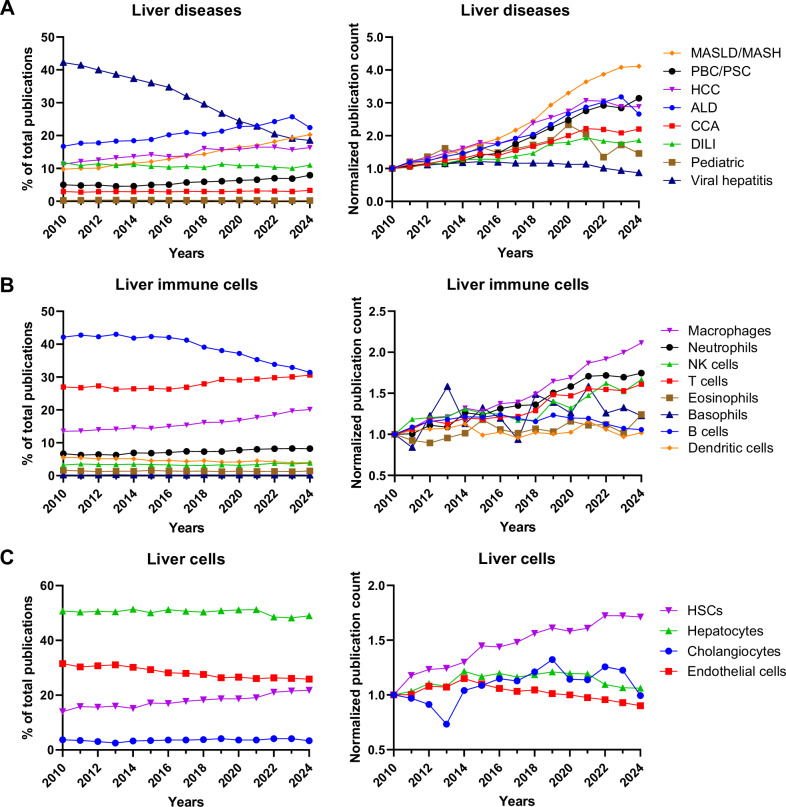
Bibliometric analysis of publications related to liver immunology in the field of hepatology from 2010–2024: Focus on disease- and cell-specific keywords. Proportional trends and normalized publication trends (from 2010 to 2024) by **A** liver diseases in hepatology, **B** immune cells in the field of hepatology, and **C** liver cells (liver parenchymal and nonparenchymal cells) in hepatology. For proportional trends, values represent the percentage of total hepatology publications attributed to each category in a given year. For normalized publication trends, each category’s annual publication count is normalized to its own 2010 level (set at 1.0) to enable comparison over time. In (**A**), we performed literature research in the Web of Science database from 2010 to 2024 using the following keywords to denote 8 categories of liver disease that are also associated with the keyword immunology: MASLD: NAFLD, MAFLD, MASLD, NASH, MASH; HCC: hepatocellular carcinoma, HCC; CCA: cholangiocarcinoma, CCA; viral hepatitis: HAV, HBV, HCV, HDV, HEV, hepatitis A virus, hepatitis B virus, hepatitis C virus, hepatitis D virus, hepatitis E virus; PBC/PSC: primary biliary cirrhosis, primary biliary cholangitis, primary sclerosing cholangitis; ALD: ALD, ARLD, AH, SAH, alcoholic liver disease, alcohol-related liver disease, alcohol-related hepatitis, alcoholic hepatitis, severe alcoholic hepatitis (“alcoholic” is a stigmatizing word, the new terminology is alcohol-related, or alcohol-associated; but we included these outdated terms in the analysis to sort older publications); DILI: DILI, acetaminophen, paracetamol in the context of liver (drug-induced liver injury); and pediatric liver diseases. In (**B**), we used the following keywords: macrophages, macrophages, Kupffer cells; T cells: T lymphocytes, Th, Treg; B lymphocytes; neutrophils; NK, NKT; eosinophils; basophils; and dendritic cells. In (**C**), we searched for hepatocytes; cholangiocytes, ductular cells, and ductular reactions; endothelial cells; and HSCs, myofibroblasts. Abbreviations: ALD, alcohol-related liver disease; CCA, cholangiocarcinoma; DILI, drug-induced liver injury; HCC, hepatocellular carcinoma; MASLD, metabolic dysfunction-associated steatotic liver disease; PBC, primary biliary cholangitis; PSC, primary sclerosing cholangitis. Courtesy of Lanlan Chen (Charité – Universitätsmedizin Berlin, Berlin, Germany & The First Hospital of Jilin University, Changchun, China)

Owing to rapid innovations in patient care options and the discovery of disease-driving mechanisms, the epidemiology of liver diseases worldwide has changed in recent years. This shift is not only a consequence of beneficial breakthroughs that enhance clinical management but also detrimental changes in lifestyle factors such as diet and physical activity. As such, the burden of viral hepatitis is lower where vaccination and effective direct antiviral drugs are available. In parallel, the incidence rates of metabolic dysfunction-associated steatotic liver disease (MASLD) and alcohol-associated liver disease (ALD) are increasing at a worrisome rate, with an estimated 30% of the general adult population showing some degree of steatotic liver disease [2, 3]. Similarly, a marked increase in the relative share of publications related to liver immunology in MASLD and ALD is observed, whereas contributions from viral hepatitis (e.g., hepatitis B and C) have steadily declined, likely attributed to therapeutic advancements (Fig. 1A).

Recent insights have extended our views on adaptive immunity, with major advances in our understanding of T and B lymphocyte biology and their intricate roles in liver cancer, as well as previously undervalued roles for myeloid immune cells and parenchymal, stromal and mesenchymal cells at all stages of liver cancer initiation and progression and in preneoplastic chronic liver diseases, as reflected in this collection review [4]. Most notably, and as reflected in this special issue, major advances have been made in the study of liver myeloid cells, particularly those of macrophages/monocytes and neutrophils (Fig. 1B) [5–7]. Our earlier views on the M1/M2 macrophage dichotomy have increased, as single-cell transcriptomics revealed a much broader and highly dynamic range of macrophage phenotypes, further refined on the basis of cell location, as demonstrated by recent spatially resolved proteomics- and transcriptomics-based analyses. A striking example of such advances is reflected by findings related to bile duct-associated macrophages observed in liver disease progression [8]. These findings highlight the concept of a macrophage niche, which evolves according to the liver development stage from embryogenesis to adulthood, or ongoing injury, as reviewed by Nusse and Kubes [6]. With the emergence of single-cell transcriptomics and spatial biology, the field has greatly advanced in dissecting the intricate roles not only of immune cells but also of other liver cells beyond hepatocytes. As such, quiescent cholangiocytes and reactive ductular cells, in addition to hepatocytes, hepatic stellate cells and endothelial cells, are receiving some attention because of their ability to orchestrate intricate cellular and interorgan crosstalk, as well as immune cell chemotaxis, adhesion, and phenotypic specialization (Fig. 1C). This topic is herein reviewed by Kostallari, Schwabe and Guillot [9].

In the near future, we anticipate that the fast-paced discovery rate of research related to liver immunology will accelerate even more, with the widespread deployment of single-cell transcriptomics technologies and the increasing use of deep learning-based algorithms (artificial intelligence). As some societies further aim at reducing the use of research animals, in vitro models are expected to become more prominent while being refined by the growing knowledge in cell biology. Related to these aspects, Rezvani’s article on human models of liver immune interactions is timely for the field of liver immunology [10]. Finally, the next years will certainly see the rise of personalized or precision medicine, in line with the development of novel technologies for targeted liver immunology interventions at the molecular level and the rise of patient cell-derived models and datasets.
